# Phosphatidylinositol-specific phospholipase C enhances epidermal penetration by *Staphylococcus aureus*

**DOI:** 10.1038/s41598-020-74692-8

**Published:** 2020-10-20

**Authors:** Yoshikazu Nakamura, Kaori Kanemaru, Madoka Shoji, Kengo Totoki, Karen Nakamura, Hidemasa Nakaminami, Keisuke Nakase, Norihisa Noguchi, Kiyoko Fukami

**Affiliations:** 1 Department of Applied Biological Science, Faculty of Science and Technology, Tokyo University of Science, Noda, Chiba Japan; 2 PRIME, Japan Agency for Medical Research and Development, Tokyo, Japan; 3 Laboratory of Genome and Biosignals, School of Life Sciences, Tokyo University of Pharmacy and Life Sciences, Hachioji, Tokyo Japan; 4 Department of Microbiology, School of Pharmacy, Tokyo University of Pharmacy and Life Sciences, Hachioji, Tokyo Japan

**Keywords:** Skin diseases, Phospholipids

## Abstract

*Staphylococcus aureus* (*S. aureus*) commonly colonizes the human skin and nostrils. However, it is also associated with a wide variety of diseases. *S. aureus* is frequently isolated from the skin of patients with atopic dermatitis (AD), and is linked to increased disease severity. *S. aureus* impairs the skin barrier and triggers inflammation through the secretion of various virulence factors. *S. aureus* secretes phosphatidylinositol-specific phospholipase C (PI-PLC), which hydrolyses phosphatidylinositol and cleaves glycosylphosphatidylinositol-anchored proteins. However, the role of *S. aureus* PI-PLC in the pathogenesis of skin diseases, including AD, remains unclear. In this study, we sought to determine the role of *S. aureus* PI-PLC in the pathogenesis of skin diseases. PI-PLC was observed to enhance the invasion and persistence of *S. aureus* in keratinocytes. Besides, PI-PLC promoted the penetration of *S. aureus* through the epidermal barrier in a mouse model of AD and the human organotypic epidermal equivalent. Furthermore, the loss of PI-PLC attenuated epidermal hyperplasia and the infiltration of Gr-1^+^ cells and CD4^+^ cells induced by *S. aureus* infection in the mouse model of AD. Collectively, these results indicate that PI-PLC eases the entry of *S. aureus* into the dermis and aggravates acanthosis and immune cell infiltration in infected skin.

## Introduction

Although *Staphylococcus aureus* (*S. aureus*) commonly colonizes human skin and nostrils, it is also associated with a wide variety of diseases or conditions, such as cellulitis, abscesses, pneumonia, endovascular disease, and toxic shock. *S. aureus* is a leading causative agent of nosocomial infections worldwide^1^. *S. aureus* is well-known for its ability to acquire resistance to various antibiotics. The emergence and spread of methicillin-resistant *S. aureus* strains, which are often multi-drug resistant, in hospital settings, has resulted in significant mortality and morbidity.

*S. aureus* is frequently isolated from the skin of patients with atopic dermatitis (AD)^2–4^. AD is characterized by skin inflammation, barrier dysfunction, and chronic pruritus. The skin microbiome in patients with AD undergoes a loss in bacterial diversity and an increase in *S. aureus* abundance^5^. Disease severity in patients with AD is linked to increased colonization of *S. aureus*^6^. *S. aureus* was observed to induce AD-like lesions in a mouse model^2^. *S. aureus* damages the skin barrier and triggers inflammation by secreting virulence factors, which include α-toxin, superantigens, toxic shock syndrome toxin-1, enterotoxins, phenol-soluble modulins, protein A, Panton–Valentine leucocidin, exfoliative toxins, and V8 serine protease^7–12^. Although determining the functions of the secretory virulence factors of *S. aureus* is important for understanding the pathogenesis of AD and identifying novel therapeutic targets, the secretory virulence factors that worsen AD are yet to be completely characterized.

*S. aureus* is able to invade and persist in keratinocytes. *S. aureus* was observed to attach to the host cell and participate in signal transduction and cytoskeletal rearrangement^13^, and was subsequently internalized by the host cells. The attachment of *S. aureus* is mediated by several cell wall-bound adhesins^14^ and secretable expanded repertoire adhesion molecules^15^. The link formed between fibronectin-binding proteins (FnBPs) and α5β1 integrins by fibronectin plays a role in *S. aureus* attachment and invasion. *S. aureus* internalization is suggested to occur through FnBPs-dependent as well as FnBPs-independent pathways in keratinocytes^16^. The extracellular adherence protein enhances staphylococcal adhesion and invasion in keratinocytes^16^. Signal transduction in host cells also plays a role in *S. aureus* invasion, and the integrin-linked kinase (ILK)‐Rac1 pathway is reported to be essential for the invasion of keratinocytes by *S. aureus*^17^. In addition, *S. aureus*-secreted lipases contribute to keratinocyte invasion^18^, which strongly suggests that lipid metabolism plays a role in host cell invasion by *S. aureus*.

*S. aureus* secretes phosphatidylinositol-specific phospholipase C (PI-PLC), which hydrolyses phosphatidylinositol (PI)^19^. Since PI is not a component of the *S. aureus* membrane^20^, PI in the host cells is the likely target of *S. aureus* PI-PLC. Besides PI, *S. aureus* PI-PLC also cleaves glycosylphosphatidylinositol (GPI)-anchored proteins from the surface of cells^19^. PI-PLCs are important virulence factors of *Listeria monocytogenes*^21^ and *Bacillus* species^22,23^. In addition, PI-PLC is observed to be expressed and secreted only by *S. aureus*, which is the most virulent human pathogen among staphylococcal species. Therefore, *S. aureus* PI-PLC likely functions as a virulence factor of *S. aureus*, as reported in relevant studies. PI-PLC was observed to promote the survival of ingested *S. aureus* in human polymorphonuclear neutrophils (PMNs) and enhance the survival of *S. aureus* against host defense mechanisms^24^. *S. aureus* PI‐PLC induces severe tissue damage, promotes pulmonary edema, and induces the progression of acute respiratory distress syndrome through the sensitization of tissues to complement activation^25^. However, the role of *S. aureus* PI-PLC in the pathogenesis of skin diseases, including AD, remains unknown. In this study, we sought to determine the role of *S. aureus* PI-PLC in the pathogenesis of skin diseases by evaluating the pathogenicity of a PI-PLC knockout strain of *S. aureus*. PI-PLC was observed to enhance the penetration of *S. aureus* through the epidermal barrier, epidermal hyperplasia, and immune cell infiltration in the skin of a mouse model of AD in an enzyme activity-dependent manner.

## Results

### Generation of PI-PLC knockout and complemented strains

We first examined whether PI-PLC is expressed and secreted by the *S. aureus* strains that colonize the skin in patients with AD. Since an antibody against PI-PLC is not commercially available, a monoclonal antibody against PI-PLC was generated by immunization with a recombinant PI-PLC protein. Western blot analysis with the generated antibody revealed that, even though the levels of PI-PLC secretion varied among different clinical isolates, PI-PLC was detected in the supernatants of all isolates cultured in the tryptic soy broth (TSB) culture medium (Supplementary Fig. S1a). To evaluate the role of PI-PLC in the pathogenesis of AD, a PI-PLC knockout *S. aureus* (Δ*plc*) and complemented strain were constructed. In the present study, the methicillin-susceptible *S. aureus* strain JCM 2874 was used as the parent strain. The Δ*plc* strain was generated by inserting a 0.9-kb group II intron into the *plc* gene (Supplementary Fig. S1b). The insertion of introns and the disruption of the *plc* gene were verified using PCR (Supplementary Fig. S1c). Western blot analysis revealed that PI-PLC was detected in the culture supernatant of the wild-type strain, whereas it was not detected in that of the Δ*plc* strain (Supplementary Fig. S1d). In addition to the Δ*plc* strain, the complemented strain was generated by reintroducing the *plc* gene into the Δ*plc* strain (designated as Δ*plc* :: *plc*). Since a previous study demonstrated the necessity of His-80 in the activity of *S. aureus* PI-PLC^25^, another strain was constructed for the complemented strain with mutated *plc* gene that encoded His-80-mutated catalytically inactive mutants of PI-PLC (designated as Δ*plc* :: *plc-MT*). This would help determine the role of PI-PLC activity. Since the anti-PI-PLC monoclonal antibody is able to recognize the catalytically inactive mutants of PI-PLC, the levels of PI-PLC in the culture supernatant of Δ*plc* :: *plc* and Δ*plc* :: *plc-MT* strain were examined. Western blot analysis revealed that PI-PLC was present in the culture supernatant of both Δ*plc* :: *plc* and Δ*plc* :: *plc-MT* strains at levels similar to those in the culture supernatant of the wild-type strain (Supplementary Fig. S1d). Next, PI-PLC activity in the culture supernatant of wild-type, Δ*plc*, Δ*plc* :: *plc*, and Δ*plc* :: *plc-MT* strains was evaluated using a colorimetric substrate. PI-PLC activity was observed in the culture supernatant of the wild-type and Δ*plc* :: *plc* strain, whereas the culture supernatant of Δ*plc* and Δ*plc* :: *plc-MT* strain did not exhibit PI-PLC activity (Supplementary Fig. S1e). Knockout and complementation of the *plc* gene did not affect the proliferation of *S. aureus* in TSB (Supplementary Fig. S1f). These results indicate that the PI-PLC knockout and complemented strains were successfully constructed, and that the loss of PI-PLC activity did not affect the proliferation of *S. aureus* under the culture conditions.

### PI-PLC enhances the persistence and proliferation of *S. aureus* within keratinocytes

**Figure 1:**
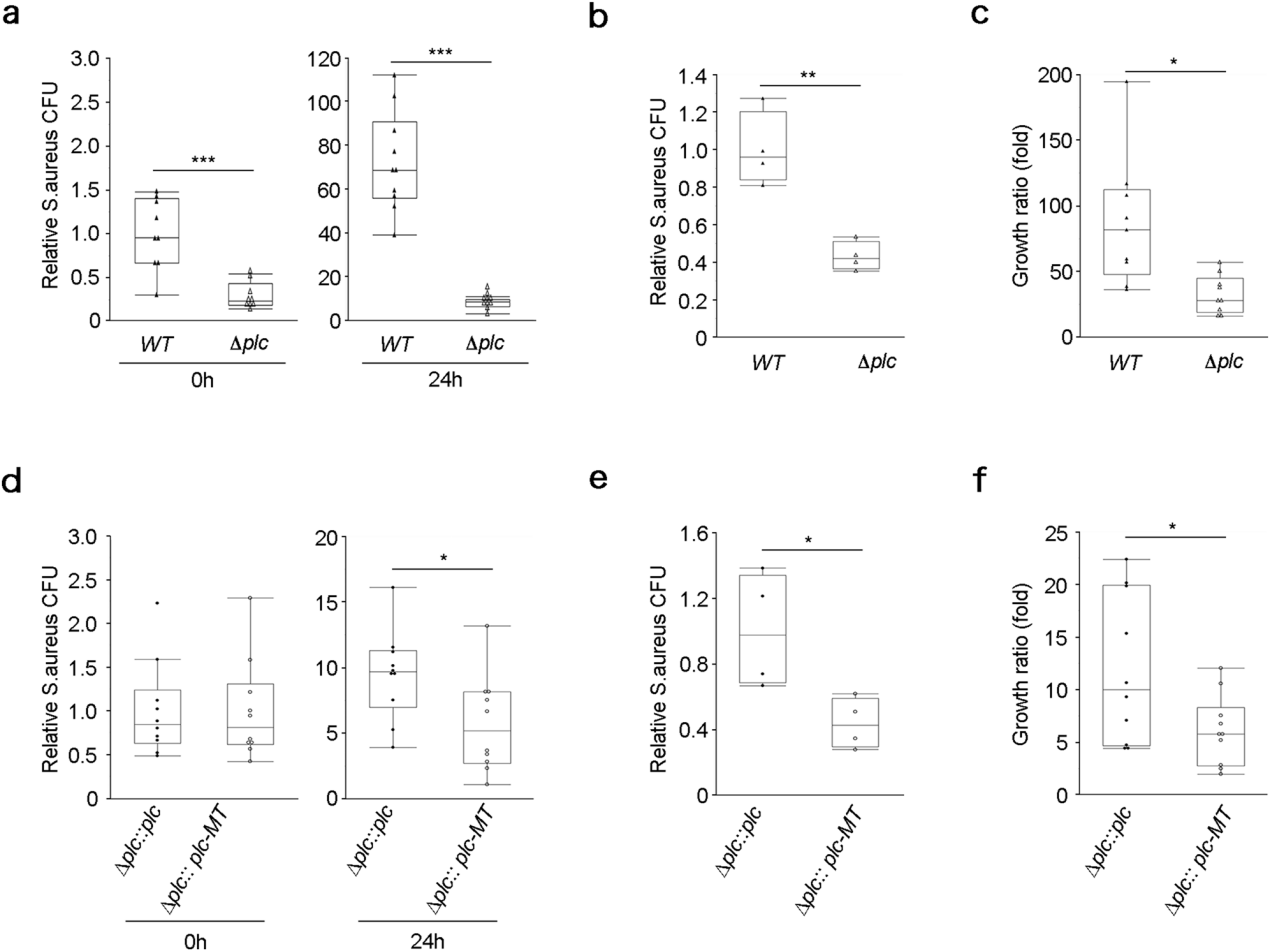
PI-PLC enhances the invasion and persistence of *Staphylococcus aureus* in human keratinocytes. Gentamicin protection assays were performed to quantify the ability of wild-type and Δ*plc* mutant strains (**a**–**c**) or Δ*plc* :: *plc* and Δ*plc* :: *plc-MT* strains (**d**–**f**) to invade and persist in HaCaT cells (**a**,**c**,**d**,**f**) and human epidermal keratinocytes (**b**,**e**). (**a**,**b**,**d**,**e**) Relative CFUs were measured immediately after infection (0 h) or at 24 h after infection [CFU of wild-type at 0 h = 1 (**a**). CFU of wild-type at 24 h = 1 (**b**). CFU of Δ*plc* :: *plc* at 0 h = 1 (**d**). CFU of Δ*plc* :: *plc* at 24 h = 1 (**e**)]. (**c**,**f**) The growth ratio of *S. aureus* was calculated by dividing CFU at 24 h after infection by CFU immediately after infection. *N* = 10 in wild-type, Δ*plc*, Δ*plc* :: *plc*, and Δ*plc* :: *plc-MT* strains (**a**,**c**,**d**,**f**). *N* = 4 in wild-type,Δ*plc*, Δ*plc* :: *plc*, and Δ*plc* :: *plc-MT* strains (**b**,**e**). Individual data values are represented by a single symbol on the box plots. Statistical significance was assessed using the Welch’s *t* test. *p < 0.05. **p < 0.01. ***p < 0.001. *S. aureus* from 10 (**a**,**c**,**d**,**f**) or 4 (**b**,**e**) distinct colonies was used for the experiments. Results are representative of two trials (**a**–**f**).

While *S. aureus* was originally considered an extracellular pathogen^26^, it has been shown to be able to invade and proliferate in mammalian cells^27–30^. Although antimicrobial peptides (AMPs) secreted from keratinocytes target extracellular *S. aureus*^31^, intracellular *S. aureus* can evade the attack. It was reported that the secretory lipases of *S. aureus* enhanced keratinocyte invasion by *S. aureus*^18^, which suggests that lipid metabolism plays a role in host cell invasion by *S. aureus*. Therefore, the role of PI-PLC in the invasion and persistence of *S. aureus* in keratinocytes was evaluated. Monolayers of HaCaT cells or human epidermal keratinocytes were infected with *S. aureus*, followed by killing of extracellular *S. aureus* using antibiotics. The number of intracellular *S. aureus* was measured by the quantification of *S. aureus* DNA and calculation of the number of relative colony forming units (CFUs). The number of intracellular Δ*plc* strains was observed to be lower than that of the wild-type strain immediately after (0 h) and 24 h after infection (Fig. 1a), which suggests that PI-PLC contributes to the invasion and persistence of *S. aureus* in HaCaT cells. The number of intracellular Δ*plc* strains in human epidermal keratinocytes was also lower than that of the wild-type strain at 24 h after infection (Fig. 1b). Although the number of relative CFUs in *S. aureus*-infected HaCaT cell lysates increased regardless of the presence of PI-PLC, the growth ratio of the Δ*plc* strain was lower than that of the wild-type strain (Fig. 1c), which suggests that PI-PLC also contributes to intracellular survival and proliferation of *S. aureus* in HaCaT cells.

Next, the role of PI-PLC activity in the survival and proliferation of *S. aureus* in HaCaT cells and human epidermal keratinocytes was examined by challenging the cells with the Δ*plc* :: *plc* and Δ*plc *:: *plc-MT* strains. The number of relative CFUs at 24 h after infection with the Δ*plc* :: *plc-MT* strain was lower than that after infection with the Δ*plc* :: *plc* strain in HaCaT cells and human epidermal keratinocytes (Fig. 1d,e). The growth ratio of the Δ*plc* :: *plc-MT* strain was also lower than that of the Δ*plc* :: *plc* strain in HaCaT cells (Fig. 1f). These results strongly suggest that PI-PLC promotes the survival and proliferation of *S. aureus* in keratinocytes in an enzyme activity-dependent manner.

### PI-PLC promotes the penetration of *S. aureus* through the epidermis

**Figure 2:**
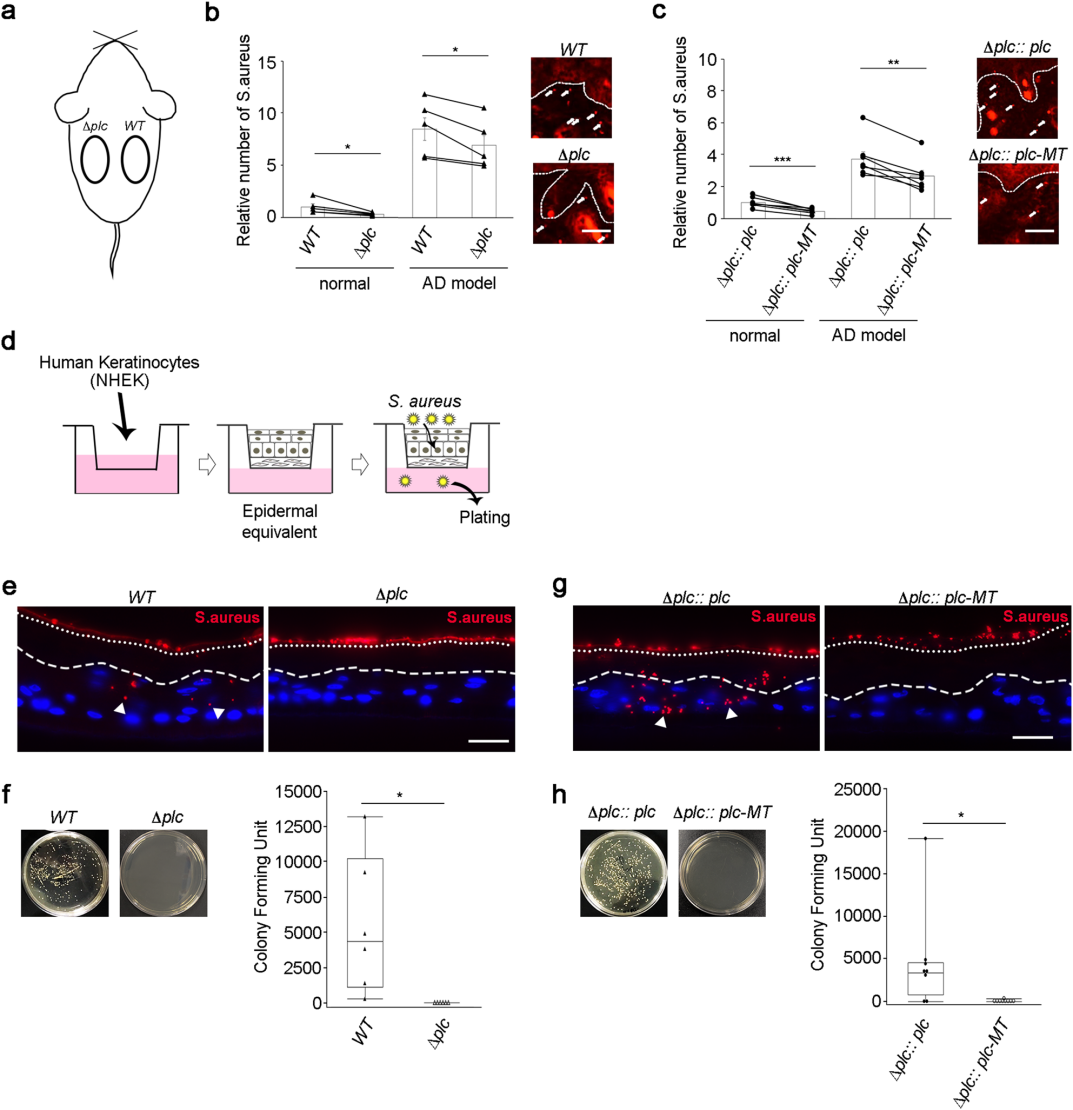
PI-PLC promotes *Staphylococcus aureus* penetration through the epidermal barrier. (**a**) Schematic depicting the location of epicutaneous infection. Wild-type and Δ*plc* strains were applied to the right and left flanks of the same mice, respectively. (**b**,**c**) The number of *S. aureus* entering the dermis of normal and AD model mice. *S. aureus* was detected by immunofluorescence with anti-*S. aureus *antibody at 4 days after epicutaneous infection by the wild-type (**b**), Δ*plc* (**b**), Δ*plc* :: *plc* (**c**), and Δ*plc *:: *plc-MT* (**c**) strains. The relative number of *S. aureus* was calculated from the average number of dermal *S. aureus* in five randomly selected 1-mm^2^ dermal areas in each mouse. *N* = 5 for wild-type and Δ*plc* strains. *N* = 7 for Δ*plc* :: *plc* and Δ*plc* :: *plc-MT* strains. The data from the right and left flank of the same mice were linked with lines. Means ± SEM. Immunofluorescence images of *S. aureus* in the dermis of AD model mice are also shown. Arrows indicate *S. aureus*. (**d**) Schematic depicting the analysis of *S. aureus* penetration through the epidermal equivalent. The depicting was generated using Adobe Photoshop and Microsoft PowerPoint. (**e**, **g**) Equivalents were stained with anti-*S. aureus* antibody (red). Keratinocyte nuclei were counterstained with Hoechst (blue). Arrowheads indicate *S. aureus* invading the equivalent. The upper and lower dotted lines denote the equivalent surface and the border between the stratum corneum and viable layers, respectively. Images are representative of six (**e**) or eight (**g**) equivalents per group. (**f**,**h**) *S. aureus* that penetrated the equivalents were detected by spreading the culture medium below the equivalent onto an agar plate. Results are representative of three technical replicates (**b**,**c**). *S. aureus* from six (**e**,**f**) or eight (**g**,**h**) distinct colonies was used. Individual data values are represented on the bar graphs or box plots. Statistical significance was assessed using the paired *t* test (**b**,**c**) and Welch’s *t* test (**f**,**h**). *p < 0.05. **p < 0.01. ***p < 0.001. Results are representative of two trials (**e**–**h**). Scale bar = 50 µm (**b**,**c**,**e**,**g**).

We next evaluated the role of PI-PLC in the entry of *S. aureus* into the dermis in normal mice, since the entry of *S. aureus* into the dermis and the subsequent aggravation of dermatitis is observed in the skin lesions in patients with AD^32^. The mice were administered an epicutaneous dose of wild-type and Δ*plc* strains by attaching filter paper discs and Finn chambers covered with surgical tapes on the shaved flank skin. To exclude potential confounding variables, the wild-type and Δ*plc* strains were administered to the right and left flanks of the same mice, respectively (Fig. 2a). The entry of *S. aureus* into the dermis decreased significantly in the skin of normal mice challenged with the Δ*plc* strain compared to that in mice challenged with the wild-type strain (Fig. 2b). In addition, the entry of *S. aureus* into the dermis decreased in mice infected with Δ*pl*c :: *plc-MT* strain compared to that in mice challenged with the Δ*plc* :: *plc* strain, which indicates that PI-PLC activity contributes to the entry of *S. aureus* into the dermis (Fig. 2c). Since the loss of PI-PLC activity led to the inhibition of *S. aureus* entry into the dermis, we hypothesized that PI-PLC may contribute to epidermal penetration by *S. aureus*. To assess this, the invasion and penetration potential of the wild-type and Δ*plc* strains in the human organotypic epidermal equivalent was evaluated (Fig. 2d). The findings of immunofluorescence experiments revealed that while the wild-type strain penetrated the epidermal equivalent, the Δ*plc* strain did not enter the epidermal equivalent and was only detected on the surface (Fig. 2e). The number of *S. aureus* penetrating the human organotypic epidermal equivalent was estimated by spreading the culture medium below the epidermal equivalent onto the agar plates. While colonies were formed when the medium below the epidermal equivalent challenged with the wild-type strain was cultured, no colonies were observed in case of the Δ*plc* strain-challenged epidermal equivalent (Fig. 2f). To evaluate the role of PI-PLC activity in epidermal invasion and penetration by *S. aureus*, the human organotypic epidermal equivalent was challenged with Δ*plc* :: *plc* and Δ*plc* :: *plc-MT* strains. While the Δ*plc* :: *plc* strain invaded and penetrated the organotypic human epidermal equivalent, the Δ*plc* :: *plc-MT* strain did not (Fig. 2g,h). These results strongly suggest that PI-PLC contributes to epidermal invasion and penetration by *S. aureus* in an enzyme activity-dependent manner.

### PI-PLC enhances epidermal thickening and immune cell infiltration induced by *S. aureus* infection in a mouse model of AD

**Figure 3:**
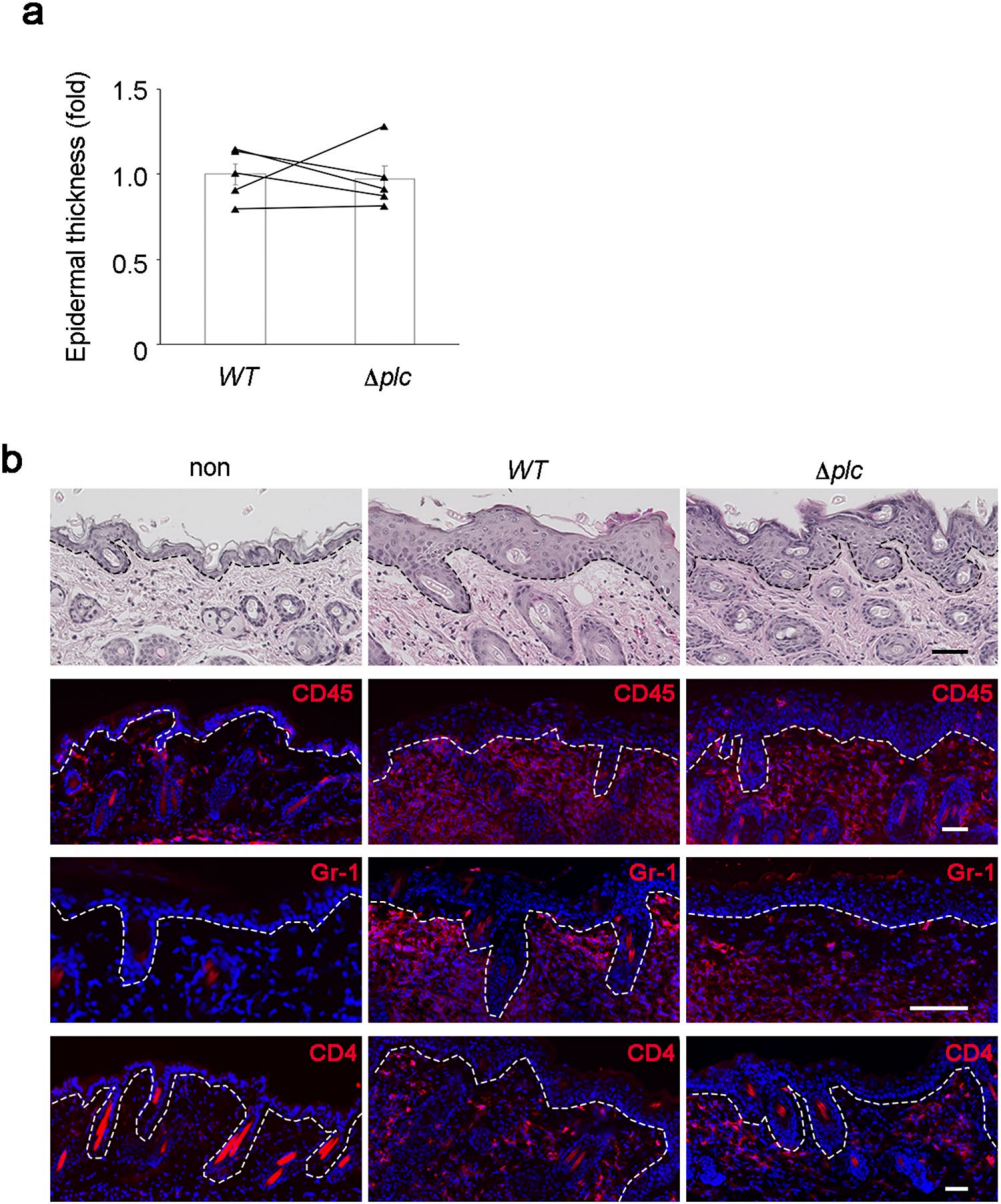
Loss of PI-PLC does not affect *Staphylococcus aureus*-induced skin inflammation in normal mice. (**a**) Epidermal thickness at 4 days after epicutaneous infection of *S. aureus*. *N* = 5 in each group. The data corresponding to the right and left flank of the same mice were linked with lines. Data are presented as the means ± SEM. Individual data values are represented by a single symbol on the bar graphs. (**b**) Staining of skin specimens with hematoxylin and eosin (top panels) or with antibodies against CD45 (red), Gr-1 (red), CD4 (red), and Hoechst (blue) at 4 days after epicutaneous infection by *S. aureus*. Staining was also performed on non-infected skin (non). Scale bar = 50 µm. Images are representative of five animals per group. Images of wild-type- and Δ*plc* mutant-infected skin in (**b**) are from the right and left flanks of the same mice, respectively. Results are representative of two technical replicates.

Since PI-PLC plays a positive role in *S. aureus* invasion and persistence in keratinocytes and penetration through the epidermis and epidermal equivalent, the effects of PI-PLC on the phenotypes of *S. aureus*-infected skin were examined. Epicutaneous infection with the wild-type and Δ*plc* strains induced mild thickening of the epidermis and infiltration of CD45^+^ leukocytes, Gr-1^+^ granulocytes, and CD4^+^ T cells. However, the loss of PI-PLC did not affect the severity of epidermal thickening, immune cell infiltration, and expression of pro-inflammatory cytokines (Fig. 3a,b, and Supplementary Fig. S2a). These results suggest that PI-PLC does not play a significant role in the mild skin inflammation induced by *S. aureus* infection in normal mice.

**Figure 4:**
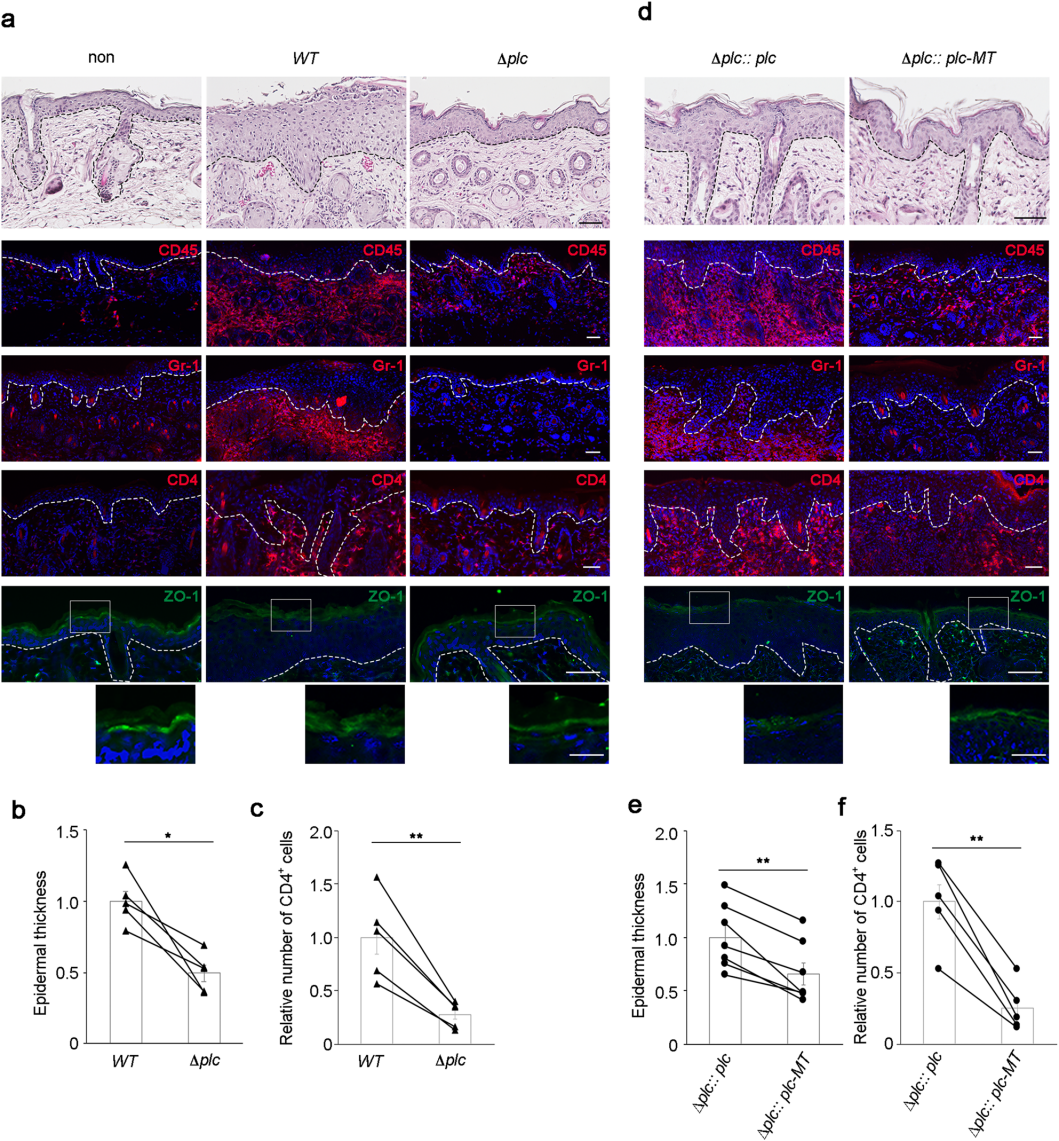
PI-PLC enhances epidermal thickening, immune cell infiltration, and ZO-1 mislocalization in *Staphylococcus aureus*-infected skin in a mouse model of AD. (**a**,**d**) Staining of skin specimens with hematoxylin and eosin (top panels) or with antibodies against CD45 (red), Gr-1 (red), CD4 (red), ZO-1 (green), and Hoechst (blue) at 4 days after epicutaneous infection by wild-type (**a**), Δ*plc *(**a**), Δ*plc* :: *plc* (**d**), and Δ*plc* :: *plc-MT* (**d**) strains. Staining was also performed on non-infected skin (non) (**a**). Scale bar = 50 µm. For ZO-1 staining, a magnified view of the indicated area (surrounded by square) is also shown. Scale bar = 20 µm. Images of wild-type- and Δ*plc* mutant-infected skin in (**a**) or Δ*plc* :: *plc*- and Δ*plc* :: *plc-MT*-infected skin in (**d**) were from the right and left flanks of the same mice, respectively. (**b**,**c**,**e**,**f**) Epidermal thickness (**b**,**e**) and number of CD4^+^ cells (**c**,**f**) at 4 days after epicutaneous infection by wild-type (**b**,**c**), Δ*plc* (**b**,**c**), Δ*plc* :: *plc* (**e**,**f**), and Δ*plc* :: *plc-MT* (**e**,**f**) strains. *N* = 5 in wild-type and Δ*plc* strains. *N* = 7 in Δ*plc* :: *plc* and Δ*plc* :: *plc-MT* strains. The data from the right and left flanks of the same mice were linked with lines. Data are presented as the means ± SEM. Individual data value are represented by a single symbol on the bar graphs. Statistical significance was assessed using the paired *t* test. *p < 0.05. **p < 0.01. Results are representative of two trials (**a**–**f**).

Given that normal mice had an intact epidermal barrier and a stable immune homeostasis, the number of *S. aureus* invading the dermis of normal mice may have been considerably low to induce clear inflammatory phenotypes. As AD mouse models have a defective physical and immune barrier, *S. aureus* was applied to the barrier-deficient skin in the AD mouse model that was generated by the topical application of a vitamin D3 analogue MC903^33^. The number of *S. aureus* that penetrated the epidermis in the AD mouse model infected with wild-type *S. aureus* was higher than that in normal mice. The entry of *S. aureus* into the dermis was inhibited significantly in skin infected with the Δ*plc* strain than in that infected with the wild-type strain (Fig. 2b). Infection by wild-type *S. aureus* induced significant epidermal thickening and immune cell infiltration (Fig. 4a,b) in the skin of the mouse model of AD. However, the Δ*plc* strain only induced mild thickening of the epidermis and low infiltration of CD45^+^ leukocytes (Fig. 4a,b). Among leukocytes, infiltration by the Gr-1^+^ granulocytes and CD4^+^ T cells was attenuated upon the loss of *S. aureus* PI-PLC (Fig. 4a,c). Regardless of the attenuated infiltration of immune cells in skin infected with the Δ*plc* strain, the expression of pro-inflammatory cytokines remained uninhibited (Supplementary Fig. S2b).

Tight junctions are one of the major physical barriers in the epidermis. Since the localization of tight junction proteins was disturbed in the lesioned skin of patients with AD^34,35^, the effect of *S. aureus* infection on the localization of ZO-1, a tight junction protein, was evaluated. Since the AD model mice only exhibited mild skin inflammation in the absence of *S. aureus* infection, ZO-1 staining was clearly observed in the upper epidermis in non-infected skin. Upon infection with the wild-type *S. aureus*, the ZO-1 staining intensity reduced and became diffused (Fig. 4a). In contrast to infection with the wild-type strain, that with the Δ*plc* strain did not affect ZO-1 localization (Fig. 4a). These results suggest that *S. aureus* PI-PLC is involved in the mislocalization of tight junction proteins in AD skin. Therefore, PI-PLC plays a critical role in *S. aureus*-induced aggravation of epidermal hyperplasia and immune cell infiltration in a mouse model of AD.

Next, PI-PLC activity was evaluated to determine its role in *S. aureus*-induced aggravation of dermatitis in a mouse model of AD. The Δ*plc* :: *plc* and Δ*plc* :: *plc-MT* strains were applied on the dorsal skin of AD model mice. The number of dermal *S. aureus* decreased significantly in skin challenged with the Δ*plc* :: *plc-MT* strains than in that challenged with the Δ*plc* :: *plc* strain (Fig. 2c). Although both Δ*plc* :: *plc* and Δ*plc* :: *plc-MT* strains induced epidermal thickening and infiltration of CD45^+^ leukocytes, Gr-1^+^ granulocytes, and CD4^+^ T cells, immune cell infiltration and epidermal thickening induced by the Δ*plc* :: *plc* strain was more prominent (Fig. 4d–f). ZO-1 localization was also disturbed in Δ*plc* :: *plc* strain-infected skin (Fig. 4d). In contrast, the Δ*plc* :: *plc-MT* strain did not induce the mislocalization of ZO-1 (Fig. 4d). These results strongly suggest that PI-PLC enhances epidermal thickening and immune cell infiltration induced by *S. aureus* infection in a mouse model of AD in an enzyme activity-dependent manner.

## Discussion

PI-PLC plays a critical role in the invasion and penetration of mouse epidermis and human organotypic epidermal equivalent by *S. aureus* . PI-PLC enhanced epidermal penetration by *S. aureus* and aggravated epidermal hyperplasia and immune cell infiltration only in the mouse model of AD, and not in normal mice. Since AD lesions are characterized by defective antimicrobial or physical barriers^36,37^, PI-PLC may facilitate epidermal penetration and skin inflammation by *S. aureus* under such conditions. Epidermal penetration by *S. aureus* is enhanced in the skin lesions of patients with AD, leading to the exacerbation of AD^32^. Since PI-PLC supports the entry of *S. aureus* into the dermis, PI-PLC may act as a promising target in AD treatment.

The mechanisms by which PI-PLC enhances the invasion of keratinocytes by *S. aureus* remain elusive. *S. aureus* secretes lipases SAL1 and SAL2 that enhance the invasion of keratinocytes by *S. aureus*^18^, which suggests that lipid metabolism plays a role in host cell invasion. PI-PLC hydrolyses GPI and removes GPI-anchored proteins from the cell surface. Since the nanoclustering of GPI-anchored proteins was reported to regulate the functions of integrin^38^, the PI-PLC-mediated shedding of GPI-anchored proteins may affect the function and clustering of α5β1 integrin and FnBP-mediated invasion by *S. aureus*. The GPI-anchored proteins CD55 and CD59 present on keratinocytes are suggested to play a role in the alleviation of AD^39^. Since *S. aureus* PI-PLC was observed to cleave CD55 and CD59 from the surface of human umbilical vein endothelial cells and mouse pneumocytes^25^, the PI-PLC-mediated shedding of CD55 and CD59 from keratinocytes might enhance epidermal penetration by *S*. *aureus*, epidermal hyperplasia, and immune cell infiltration in *S. aureus*-infected skin in AD model mice.

Although keratinocyte-derived AMPs kill extracellular *S. aureus* in the epidermis and inhibit epidermal penetration by *S. aureus*, AMPs are less effective in combating intracellular *S. aureus*. Since PI-PLC contributes to the intracellular survival and proliferation of *S. aureus* in HaCaT cells, PI-PLC may protect *S. aureus* from AMPs and enhance epidermal penetration by supporting the intracellular persistence of *S. aureus*. Besides AMPs, *S. aureus* is able to evade antibiotic eradication by invasion and persistent colonization in cells such as keratinocytes^40^. Therefore, PI-PLC inhibition may enhance the efficiency of antibiotic treatment. Another study reported that the loss of PI-PLC reduced the viability of *S. aureus* in human blood and PMNs^24^. Given that PI-PLC supports the intracellular persistence of *S. aureus* in phagocytes and keratinocytes, PI-PLC may act as a therapeutic target for skin diseases such as AD as well as for other infectious diseases.

PI-PLC in *Listeria monocytogenes* is known to contribute to phagosomal escape, possibly by hydrolyzing PI in the phagosome membrane^41,42^; therefore, *S. aureus* PI-PLC may enhance its intracellular persistence by supporting endosomal escape in keratinocytes via mechanisms similar to those followed by *Listeria monocytogenes* PI-PLC.*.*

The Δ*plc* :: *plc* strain exhibited lower relative CFU and growth ratio compared to the wild-type strain. Although the exact reason for the lower relative CFU and growth ratio of the Δ*plc* :: *plc* strain was not clear, chloramphenicol selection for the generation of transformants harboring the *plc* gene may affect the character of the *S. aureus* strain. Nonetheless, the relative CFU and growth ratio of the Δ*plc* :: *plc* strain were higher than those of the Δ*plc* :: *plc-MT* strain, which indicates that the enzyme activity of PI-PLC plays a positive role in the persistence and proliferation of *S. aureus* in keratinocytes.

PI-PLC secreted by intracellular *S. aureus* may disturb PI metabolism in keratinocytes. Exogenous PI metabolism by *S. aureus* PI-PLC also affects the concentration of the phosphorylated forms of PI (PIPs). Since PIPs metabolism plays a crucial role in maintenance of skin barrier integrity^43^, PI-PLC may damage the epidermal barrier by affecting the concentration and metabolism of PIPs in keratinocytes.

Stratum corneum and tight junctions are physical barriers of the epidermis^44,45^ that prevent the penetration of *S. aureus* through the epidermis. Since *S. aureus* infection was observed to disturb the localization of the tight junction protein ZO-1 in a PI-PLC activity-dependent manner, PI-PLC may enhance epidermal penetration by *S. aureus* by disturbing the tight junction barrier.

GPI-anchored proteins are located in cholesterol/sphingolipid-rich membrane domains, known as lipid rafts. Lipid rafts serve as platforms for signal transduction proteins, including GPI-anchored proteins. Since keratinocytes with damaged lipid rafts exhibit gene expression patterns similar to those observed in AD biopsies, lipid raft dysfunction is suggested to be related to AD^46^. Given that PI-PLC hydrolyses and removes GPI-anchored proteins from the cell surface, PI-PLC may aggravate AD by perturbing lipid raft-mediated signal transduction. Detailed analysis of the changes in composition and localization that occur in phospholipids and GPI-anchored proteins as a result of *S. aureus* infection will help clarify the mechanisms underlying PI-PLC-mediated epidermal penetration by *S. aureus*.

Collectively, the findings of this study indicate that PI-PLC promotes epidermal penetration by *S. aureus* and enhances *S. aureus*-induced epidermal hyperplasia and immune cell infiltration. Given that PI-PLC enhances epidermal penetration by *S. aureus*, it might induce systemic infection by inducing the entry of *S. aureus* through the skin surface. In this study, we used JCM 2874 as the parent strain. Future studies are necessary to clarify the role of PI-PLC in pathogenesis by a virulent strain such as MRSA USA300 and the exact mechanisms by which PI-PLC enhances epidermal penetration and intracellular persistence of *S. aureus*.

## Methods

### Bacterial strains, media, and growth conditions

*S. aureus* strains were cultured under aeration in TSB (BD, Franklin Lakes, NJ, USA) or tryptic soy agar (TSA). Clinical isolates were collected from patients with AD admitted in a hospital in Tokyo, Japan, from 2010 to 2011. All isolates were obtained from pus samples. All methods used in this study were performed in accordance with the relevant guidelines and regulations in this research field, and the study protocol was approved by the Tokyo University of Pharmacy and Life Sciences Ethics Committee (#12-08). Informed consent was not required from the patients because the study did not involve clinical interactions. To assess growth in TSB, overnight cultures of *S. aureus* JCM 2874 (ATCC 29213) were diluted in TSB (1:200). The strain was then cultured at 37 °C under shaking conditions, and absorbance was measured at 600 nm. The PI-PLC knockout Δ*plc* strain was constructed from JCM 2874 by the insertion of a group II intron into *plc* using the primer design software and plasmid system provided with the TargeTron Gene Knockout System (Sigma-Aldrich, St. Louis, MO, USA) according to the manufacturer’s instructions. Intron insertion was confirmed by performing PCR with two *plc*‐specific primers: 5′-ATGAGTGGTTGGTATCATTC-3′ and 5′-CACTTACGATATCATCATATCC-3′ (Supplementary Fig. S1b, S1c, S3a).

### Cloning of *plc*

*plc* was amplified by PCR and the products were ligated into the polylinker segment of pTZN10 and cloned into *Escherichia coli* DH5α^47^. The pTZN10 plasmids carrying *plc* were introduced into *S. aureus* RN4220 and then into JCM 2874Δ*plc* by electroporation^48^. The transformants carrying the *plc* gene were directly selected using TSA supplemented with 10 µg/mL chloramphenicol. The inserts of the plasmid were confirmed by DNA sequencing^47^.

### Western blot analysis

The culture supernatants of various *S. aureus* strains were subjected to SDS-PAGE and subsequently transferred onto PVDF membranes. The membranes were blocked with 10% skim milk and probed with an anti-PI-PLC antibody, followed by incubation with an HRP‐conjugated secondary antibody (Dako, Glostrup, Denmark). Images were recorded using C‐DiGit (LI‐COR Biosciences, Lincoln, NE, USA) or LuminoGraph I (ATTO, Tokyo, Japan) (Supplementary Fig. S3b-c). The culture supernatants were obtained by culturing various strains of *S. aureus* overnight in TSB at 37 °C.

### PI-PLC activity assay

The activity of PI-PLC was determined using the artificial substrate 5-bromo-4-chloro-3-indolyl-myo-inositol 1-phosphate ammonium salt (Sigma-Aldrich). The reaction mixtures consisted of 90 mM Tris–HCl (pH 6.8), 2 mM substrate, 0.1% Triton X-100, and 10% culture supernatant. The culture supernatants were obtained by culturing various strains of *S. aureus* overnight in TSB at 37 °C. The reaction mixtures were placed in a 96-well plate and incubated for 7 h at 37 °C, and the absorbance was measured at 650 nm using a microplate reader SH-9000Lab (Corona Electric, Ibaraki, Japan).

### Gentamicin protection assay

To evaluate the levels of intracellular survival of JCM2874 wild-type, Δ*plc*, Δ*plc* :: *plc*, and Δ*plc* :: *plc-MT* strains, HaCaT cells and human epidermal keratinocytes HPEK (CELLnTEC, Bern, Switzerland) were infected for 2 h at an MOI of 10 and 50, respectively. The cells were washed twice with PBS. HaCaT cells were cultured in DMEM-high glucose (Thermo Fisher Scientific, Wilmington, DE, USA) supplemented with 10% fetal bovine serum and containing gentamicin (Wako, Osaka, Japan) at a final concentration of 0.1 mg/mL for 1 h to kill extracellular bacteria^47^. HPEK cells were cultured in CnT-PR containing gentamicin (Wako) at a final concentration of 0.1 mg/mL for 1 h to kill extracellular bacteria. Next, the infected HaCaT and HPEK cells were cultured for an additional 24 h to evaluate the persistence of *S. aureus*. Immediately after gentamicin treatment or after 24 h of culture, the infected cells were washed twice in PBS and total genomic DNA was extracted using DNeasy Blood & Tissue Kit (QIAGEN, Hilden, Germany). The abundance of *S. aureus* DNA in the eluate was determined by quantitative real-time PCR using THUNDERBIRD SYBR qPCR Mix (TOYOBO, Osaka, Japan) with species-specific primers for the *S. aureus* *gmk* gene^49^. The primers used for real-time PCR were 5′-TGCTGAATATGTAGGCAACTATTATG-3′ and 5′-CTAACTTGCTTTGCACCTTCTACT-3′. To determine the relative CFU, a standard curve was constructed using readings corresponding to the genomic DNA extracted from an *S. aureus* culture with known number of CFUs.

### In vitro invasion and penetration assays in human organotypic epidermal culture

NHEK (KURABO, Osaka, Japan) were cultured in HuMedia-KG2 (KURABO) supplemented with insulin, bovine pituitary extract, epidermal growth factor, hydrocortisone, kanamycin, and amphotericin B. Cells from the second passage were used for the experiments. The cells were seeded onto cell culture inserts (Millipore, Billerica, MA, USA) and cultured overnight in an assay medium (Japan Tissue Engineering, Aichi, Japan). Next, the cultures were raised to the air–liquid interface and cultured in the assay medium for 6 days to form a multi-layered epidermis. JCM2874 wild-type, Δ*plc*, Δ*plc* :: *plc*, and Δ*plc* :: *plc-MT* strains were cultured in TSB, washed with PBS, and resuspended in PBS. Fifty microliters of *S. aureus* (1 × 10^7^ CFU) was applied on the central surface of the human organotypic epidermal equivalent using a micropipette. The epidermal equivalent was harvested at 15 h after the *S. aureus* challenge, mounted on OCT, and cryo-sectioned. The entry of *S. aureus* into the epidermal equivalent was monitored by immunostaining with an anti-*S. aureus* antibody (Abcam, Cambridge, UK). The culture medium of the epidermal equivalent was harvested and plated on TSA. The number of colonies formed were counted.

### *S. aureus* infection in the normal and AD mouse model

To induce AD-like skin inflammation, 1 nmol of MC903 (Sigma-Aldrich) in 30 µL ethanol was painted on the dorsal and flank skin of 4 week-old female Balb/c mice daily for 15 days^20^. The dorsal and flank skin surfaces of normal or AD model mice (6 week-old) were shaved at least 24 h before infection. JCM2874 wild-type, Δ*plc*, Δ*plc* :: *plc*, and Δ*plc* :: *plc-MT* strains were cultured in TSB, washed with PBS, and resuspended in PBS. After careful disinfection of the skin surfaces, filter paper discs and Finn chambers (SmartPractice, Phoenix, AZ, USA) containing each *S. aureus* strain (1 × 10^8^ CFU in 30 µL) were applied to the skin. Next, the filter paper discs and Finn chambers were covered with surgical tapes. After 96 h, the filter paper discs and Finn chambers were removed and the skin sample was harvested for the experiments. All animal studies were approved by the animal experiments review board of Tokyo University of Pharmacy and Life Sciences. All animal experiments were performed in accordance with relevant guidelines and regulations.

### Histological, immunohistochemical, and immunofluorescence assays

For hematoxylin and eosin (H&E) staining, the skin tissue was fixed with 4% paraformaldehyde in PBS and embedded in paraffin. Five‐micrometre‐thick paraffin sections were cut and used for staining. The epidermal thickness was calculated from the images of the H&E-stained skin sections using Image J (NIH). Immunofluorescence analysis of CD45 (eBioscience, San Diego, CA, USA), Gr-1 (BD), CD4 (BD), *S. aureus* (Abcam), and ZO-1 (Thermo Fisher Scientific) was performed using the frozen sections. These frozen sections were fixed with 4% paraformaldehyde in PBS, blocked with TNB (PerkinElmer, Boston, MA, USA), probed with primary antibodies, and then incubated with Alexa Fluor‐conjugated secondary antibodies (Thermo Fisher Scientific). Counterstaining was performed using Hoechst 33342 (Thermo Fisher Scientific). The sections were imaged using a BZ‐X700 microscope (Keyence, Osaka, Japan).

### RNA extraction and real-time RT-PCR

Total RNA was isolated from the whole skin specimens of mice using the RNeasy Mini Kit (QIAGEN). Template cDNA was synthesized from total RNA using the ReverTra Ace qPCR RT Kit (TOYOBO) according to the manufacturer's instructions. Real-time PCR was performed using the THUNDERBIRD SYBR qPCR Mix (TOYOBO) and CFX96 thermocycler (Bio‐Rad). The primer sequences used are listed in Supplementary Table S1.

### Statistical analysis

Results are expressed in terms of mean ± SEM. Statistical analyses were performed using a two-sided Welch's t-test. For data on *S. aureus* infection in mouse skin, statistical analyses were performed using a paired *t*-test. Tukey–Kramer method was used to adjust for multiple comparison. p-value < 0.05 was used to determine the statistical significance.

## Supplementary information

Supplementary Information
