# Supplementary material for: Phosphatidylinositol-specific phospholipase C enhances epidermal penetration by Staphylococcus aureus
Source: Sci Rep. 2020 Oct 20;10:17845. doi: 10.1038/s41598-020-74692-8 (PMC7575579; doi:10.1038/s41598-020-74692-8)
Supplement: Supplementary file 1 — Supplementary Information [file 41598_2020_74692_MOESM1_ESM.pdf]

Supplementary Information

Phosphatidylinositol-specific phospholipase C enhances epidermal penetration by *Staphylococcus aureus*

Yoshikazu Nakamura[a,b,c,*], Kaori Kanemaru[a,c], Madoka Shoji[c], Kengo Totoki[c], Karen Nakamura[c], Hidemasa Nakaminami[d], Keisuke Nakase[d], Norihisa Noguchi[d], Kiyoko Fukami[c,*]

Supplementary Figures and Table:

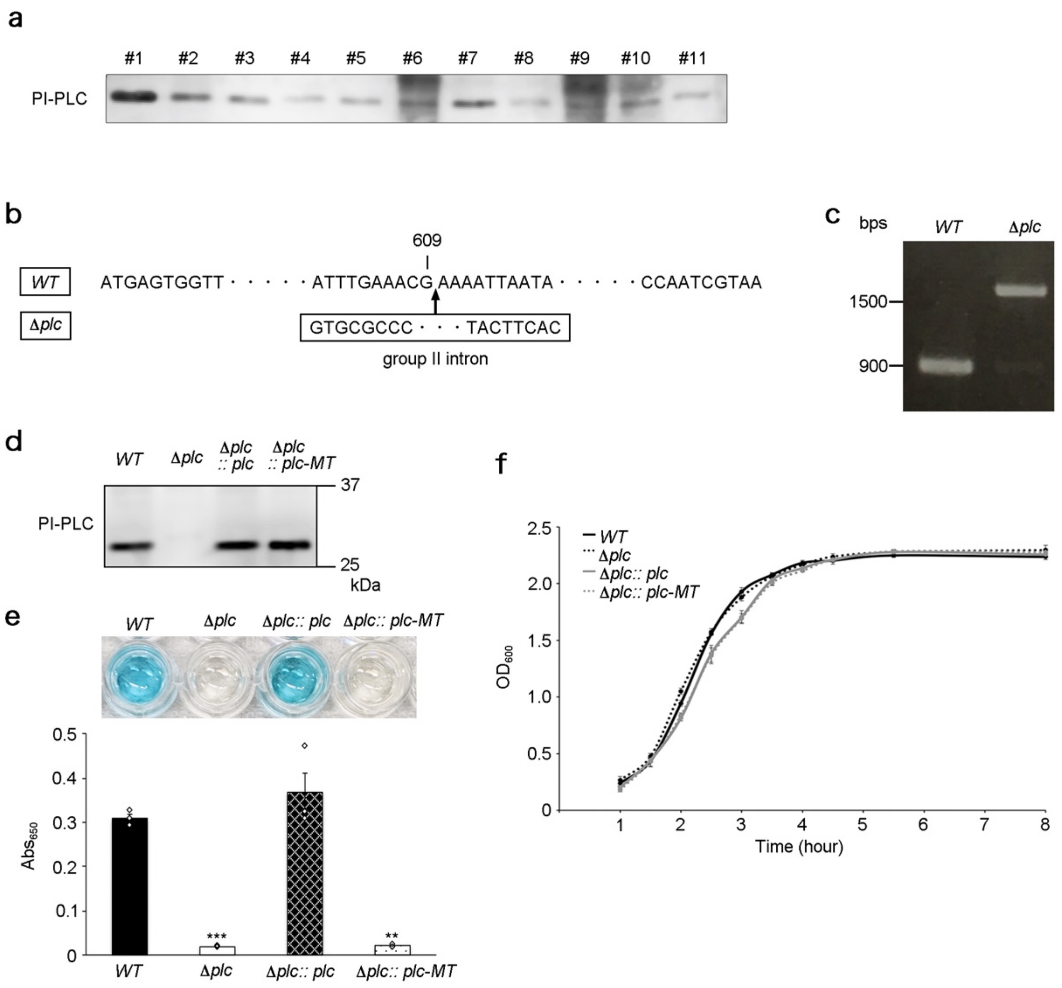

Supplementary Figure S1. Generation of PI-PLC knockout and complemented strains

(a) Western blot analysis of PI-PLC using culture supernatants of *S. aureus* isolates from 11 patients with AD (#1-#11). **(b)** Strategy for gene disruption. Type II introns were inserted between the nucleotides 609 and 610 in the sense strand of the coding sequence of *plc* to disrupt it. **(c)** Disruption of *plc* was confirmed by amplification of the DNA fragments containing the intron insertion sites by PCR. **(d)** Western blot analysis of PI-PLC using culture supernatants of wild-type, Δ*plc*, Δ*plc* :: *plc*, and Δ*plc* :: *plc-MT* strains using a monoclonal antibody against PI-PLC. **(e)** The enzymatic

activity of PI-PLC was measured using 5-bromo-4-chloro-3-indolyl-myo-inositol 1-phosphate. PI-PLC activities of the overnight culture supernatants were determined by measuring the absorbance at 650 nm. $N = 3$ in each group. Data are expressed in terms of mean ± SEM. Individual data values are represented by a single symbol on the bar graphs. Statistical significance was assessed using Tukey's test. $^{**}p < 0.01$. $^{***}p < 0.001$. **(f)** *S. aureus* was cultured at 37 °C under shaking conditions, and absorbance was measured at 600 nm at 30 minute intervals between 1 and 6 h and at 8 h of culture. *S. aureus* from three independent colonies for each strain were used for the experiments. Three biological replicates were performed (**a**, **c**-**f**).

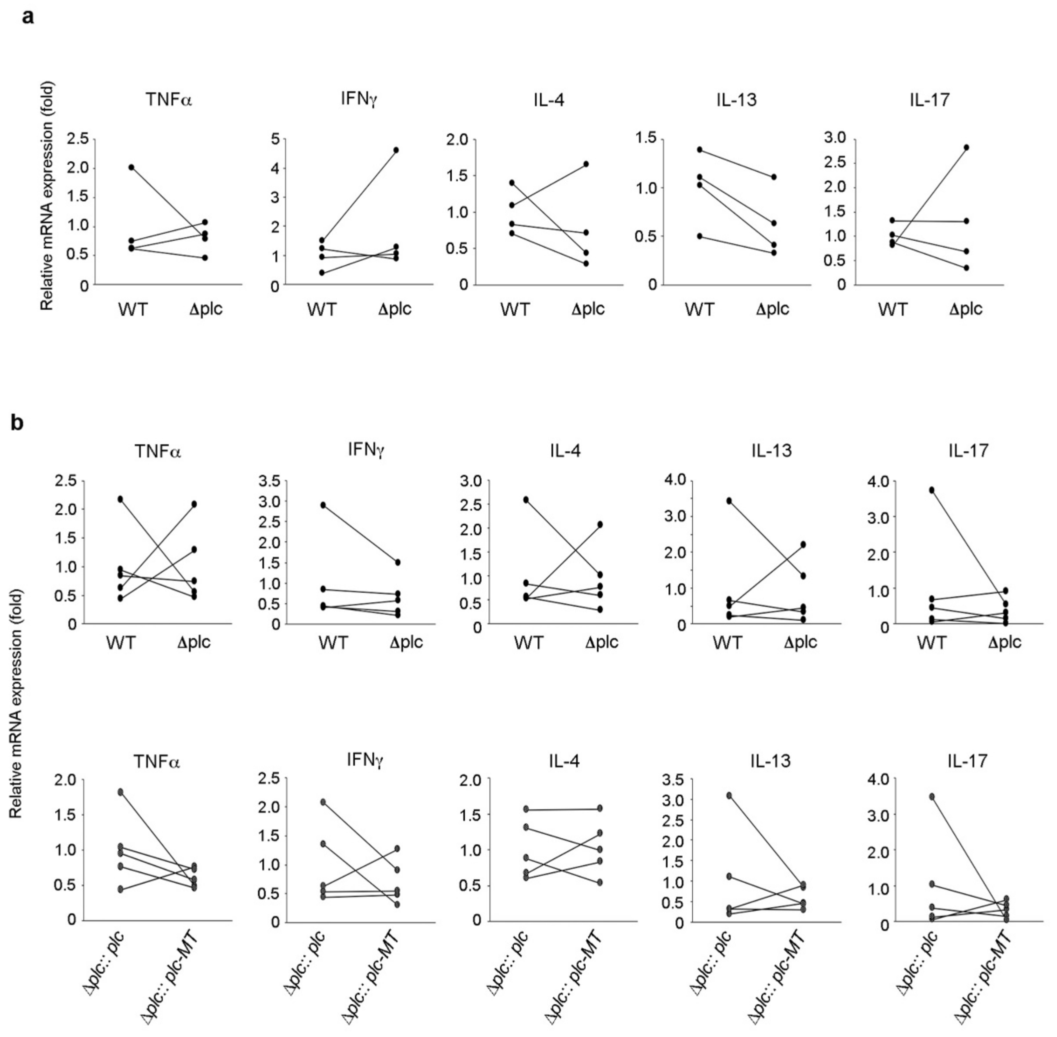

Supplementary Figure S2. PI-PLC did not affect expression of pro-infalammatory cytokines in normal and AD model mice infected with *Staphylococcus aureus*

(**a, b**) TNFα, IFNγ, IL-4, IL-13, and IL-17 mRNA expression in the skin of normal (**a**) or AD model mice (**b**) at 4 days after epicutaneous infection by wild-type, Δ*plc*, Δ*plc* :: *plc*, and Δ*plc* :: *plc-MT* strains. The values are normalized to the mRNA levels of glyceraldehyde 3-phosphate dehydrogenase. *N* = 4 in each group (**a**) and *N* = 5 in each group (**b**). The data from the right and left flanks of the same mice were linked with lines.

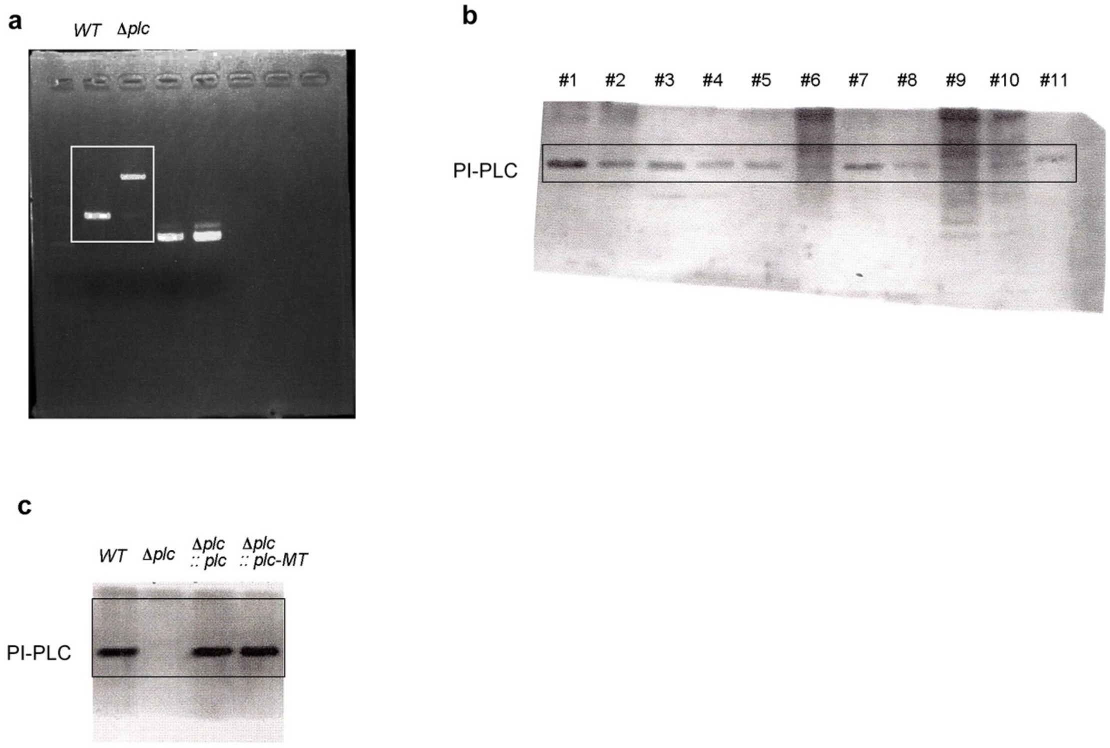

Supplementary Figure S3. Images of the original length blots and gel

(a) Western blot analysis of PI-PLC in culture supernatants of *S. aureus* isolates from 11 patients with AD (#1-#11). **(b)** Disruption of *plc* was confirmed by amplification of the DNA fragments containing the intron insertion sites by PCR. **(c)** Western blot analysis of PI-PLC in culture supernatants of wild-type, Δ*plc*, Δ*plc* :: *plc*, and Δ*plc* :: *plc-MT* strains using a monoclonal antibody against PI-PLC.

Supplementary Table S1

Primer List

PI-PLC	Forward	5′-ATGAGTGGTTGGTATCATTC-3’
	Reverse	5′-CACTTACGATATCATCATATCC-3’
hGAPDH	Forward	5′-GTCTTCTCCACCATGGAGAAGGGT-3’
	Reverse	5′-CCATGCCAGTGAGCTCCCGTTCA-3’
mTNFα	Forward	5′-CTCTTCTGCCTGCTGCACTT-3’
	Reverse	5′-GGCTACAGGCTTGTCACTC-3’
mIFNγ	Forward	5′-GCTTTAACAGCAGGCCAGAC-3′
	Reverse	5′-GGAAGCACCAGGTGTCAAGT-3’
mIL-4	Forward	5′-CATGGGAAAACTCCATGCTT-3’
	Reverse	5′-ATGAATCCAGGCATCGAAAA-3’
mIL-13	Forward	5′-TCTTGCTTGCCTTGGTGGTCTCGC-3’
	Reverse	5′-GATGGCATTGCAATTGGAGATGTTG-3’
mIL-17	Forward	5′-GTTCCACGTCACCCTGGAC-3’
	Reverse	5′-CTTTCCCTCCGCATTGACAC-3’
mGAPDH	Forward	5′-CCATGCCATCACTGCCACCC-3’
	Reverse	5′-TGTCATCATACTTGGCAGGTTTC-3’
